# Beyond right or wrong: More effective feedback for formative multiple-choice tests

**DOI:** 10.1007/s40037-020-00606-z

**Published:** 2020-08-12

**Authors:** Anna Ryan, Terry Judd, David Swanson, Douglas P. Larsen, Simone Elliott, Katina Tzanetos, Kulamakan Kulasegaram

**Affiliations:** 1 Department of Medical Education, University of Melbourne, Melbourne, Victoria Australia; 2 American Board of Medical Specialties, Chicago, Illinois USA; 3 Department of Neurology, Washington University in St. Louis School of Medicine, St. Louis, USA; 4 Department of Medicine & MD Program, University of Toronto, Toronto, Ontario Canada; 5 Department of Family & Community Medicine & The Wilson Centre, University of Toronto, Toronto, Ontario Canada

**Keywords:** Feedback, Test enhanced learning, Multiple choice questions, Practice tests, Retention, Transfer, Medical education

## Abstract

**Introduction:**

The role of feedback in test-enhanced learning is an understudied area that has the potential to improve student learning. This study investigates the influence of different forms of post-test feedback on retention and transfer of biomedical knowledge within a test-enhanced learning framework.

**Methods:**

64 participants from a Canadian and an Australian medical school sat two single-best-answer formative multiple choice tests one week apart. We compared the effects of conceptually focused, response-oriented, and simple right/wrong feedback on a learner’s ability to correctly answer new (transfer) questions. On the first test occasion, participants received parent items with feedback, and then attempted items closely related (near transfer) to and more distant (far transfer) from parent items. In a repeat test at 1 week, participants were given different near and far transfer versions of parent items. Feedback type, and near and far transfer items were randomized within and across participants.

**Results:**

Analysis demonstrated that response-oriented and conceptually focused feedback were superior to traditional right/wrong feedback for both types of transfer tasks and in both immediate and final retention test performance. However, there was no statistically significant difference between response-orientated and conceptually focused groups on near or far transfer problems, nor any differences in performance between our initial test occasion and the retention test 1 week later. As with most studies of transfer, participants’ far transfer scores were lower than for near transfer.

**Discussion:**

Right/wrong feedback appears to have limited potential to augment test-enhanced learning. Our work suggests that item-level feedback and feedback that identifies and elaborates on key conceptual knowledge are two important areas for future research on learning, retention and transfer.

## Introduction

Medical students are expected to learn, understand, retain and transfer a huge volume of information over the course of their studies. After graduation and throughout their career, they call on this knowledge reservoir to provide care to patients and refine and extend it with new and updated information gained from the medical literature, their clinical experiences, and from their growing familiarity with the healthcare system. Students and doctors alike draw on a range of learning strategies to cope with this volume of information. Formative testing – so called test-enhanced learning – is among the most effective of these and has consistently been shown to improve learning and retention of content across educational contexts [1–3] including medical education [4, 5]. Moreover, practice testing with feedback is consistently more effective than practice testing without feedback, [6] with feedback increasing the likelihood of correct answers during follow-up testing [7]. While the provision of feedback is clearly beneficial, what remains unclear is what form this feedback should take to optimize learning. For feedback to be most effective it should promote learning beyond simply retention. That is, it should ideally promote transfer of learning to new problems and align learners with expert-like schemas for clinical reasoning.

It is unclear whether current post-assessment feedback for multiple choice question (MCQ) tests improves transfer to novel problems. The most common feedback given after tests using the MCQ format is a simple indication of whether the chosen response is right or wrong. However, educators often emphasize to students that successful learning (and successful clinical problem solving) requires the application of underlying knowledge of the basic biomedical and clinical sciences to the patient context, rather than a pure memorization of facts. If that is true, then feedback is likely to be more effective if it helps learners to understand foundational underlying principles behind the patient problem represented in the question. Truly effective feedback should also ‘feedforward’ to enable students to extend and apply their knowledge to new problems [8], i.e., to stimulate transfer of learning. To promote transfer, more extensive and purposeful feedback may be necessary. Transfer occurs when conceptual knowledge structures are elaborated to facilitate a learner’s understanding of the underlying deep structure of a problem or the learning material [9, 10]. That is, beyond right and wrong, a learner must be able to appraise ‘why’ the answer is correct and other options are incorrect.

One such approach is to provide more detailed feedback highlighting why various options are correct or incorrect (e.g. C is correct because … versus B can’t be correct because … etc.). This comparative approach across response options can help learners to bridge knowledge gaps but may also be limited by an overt focus on specific details of the individual item. This form of feedback should help learners to transfer any gained knowledge to new but similar items (i.e. near transfer) but, due to its specificity, may not potentiate far transfer (i.e. transfer to novel problems in unfamiliar contexts, or novel and structurally related problems) [11]. However, the application and extension of knowledge necessary for far transfer might be best supported by feedback that specifically targets ‘conceptual’ understanding and promotes the development of problem-solving schemas [9, 12, 13].

That feedback plays a key role in promoting learning and retention of knowledge, as well as having the potential to extend the transfer of this knowledge to new problems or situations, seems clear. What is less clear is issues such as what form this feedback should take, how it should be presented, and how each of these relate to the assessment task. This paper describes an initial step towards exploring these issues, through an experimental investigation of whether and to what degree three different types of feedback – simple right/wrong, response-oriented, and detailed conceptually focused feedback – promote knowledge transfer in MCQ-based assessments. While this study was primarily exploratory rather than confirmatory, our basic working hypotheses were that elaborated types of feedback would be superior to simple right/wrong feedback for both near and far transfer and that conceptually focused feedback would be superior for promoting far transfer of knowledge.

## Methods

### Study design

This experiment employed a within-subjects experimental design. Participants in the study sat an initial MCQ test (consisting of 17 items called parent items) with feedback presented upon submission of a response for each item. The feedback was provided in one of three forms:

1. Simple identification of the correct response (right/wrong feedback);
2. Brief explanations of why each option was correct or incorrect (response-oriented feedback);
3. More detailed discussion of the correct response designed to promote transfer (conceptually focused feedback).

Participants were assigned to one of six different between-subjects blocks in which we counterbalanced the manipulations applied (conceptually focused vs. response-oriented vs. right/wrong) to particular sets of items. They then completed an immediate post-test of near and far transfer versions of the parent item with no additional feedback. One week after the initial test, participants completed a retention test with different but related near and far transfer versions of the parent items with no subject seeing the identical item twice. This design is schematically represented in Tab. 1.

**Table 1:** Schematic representation of the participant blocks (Groups A–F) used in the study design

	Parent items	Feedback sequence	Items at immediate post-test	Items at 1 week retention test
Group A	1–17	X	Items 18–34 Items 35–52	Items 53–68 Items 69–85
Group B	1–17	Y	Items 18–34 Items 35–52	Items 53–68 Items 69–85
Group C	1–17	Z	Items 18–34 Items 35–52	Items 53–68 Items 69–85
Group D	1–17	X	Items 53–68 Items 69–85	Items 18–34 Items 35–52
Group E	1–17	Y	Items 53–68 Items 69–85	Items 18–34 Items 35-52
Group F	1–17	Z	Items 53–68 Items 69–85	Items 18–34 Items 35–52

Feedback sequence X received conceptually focused feedback on the first 6 parent items, then response-oriented feedback on the next 6 parent items, then right/wrong feedback on the last 5 parent items

Feedback sequence Y received response-oriented feedback on the first 6 parent items, then right/wrong feedback on the next 6 parent items, then conceptually focused feedback on the last 5 parent items.

Feedback sequence Z received right/wrong feedback on the first 6 parent items, then conceptually focused on the next 6 parent items, then response-oriented feedback on the last 5 parent items

Tests were delivered electronically. Time stamps for screen view changes allowed collection of basic information on the amount of time participants spent responding to each item and attending to each type of feedback.

### Setting

Participants were year‑2 students at the University of Melbourne and the University of Toronto medical schools. Both schools use multiple-choice testing extensively within their medical programs.

Year‑2 medical students at both sites were invited to participate through an initial recruitment presentation in class followed by an email invitation. Participation in the study was voluntary, and written consent was gained from all participants. The study was approved by the relevant Human Research Ethics committee at each location (University of Melbourne Ethics ID 1749838 & University of Toronto Ethics ID 00034970), and the work was carried out in accordance with the Declaration of Helsinki.

The University of Melbourne has a 4-year graduate entry medical program with roughly 360 students in each year-level cohort. The majority of students enter the course directly following a biomedicine or bioscience-based undergraduate degree. The medical curriculum includes a campus-based first year with an emphasis on medical sciences, followed by 3 years in clinical settings. The University of Toronto medical program is a 4-year post-bachelor program, with approximately 260 students in each year level. The program is pre-clinical and campus-based for the first 2 years, followed by 2 clinical years organized into block rotations.

The Melbourne-based test administrations took place in late February and early March 2018, when participants were in the early stages of their second year of study. The Toronto-based tests took place between January and May 2018 when participants were about halfway through their second year.

### Materials

To test our hypotheses, we began by identifying areas of clinical reasoning that can prove difficult for early clinical learners. We then identified the underlying foundational concepts that are applied when solving these diagnostic challenges. Through a process of consultation with clinicians and MCQ-writing experts, we created 17 parent items, each centered around these foundational concepts. All items were type A single-best-answer multiple choice questions focused on application of basic science knowledge to clinical situations. For each parent item we developed four related items. We classified the closely related variants as near transfer items and the more distantly related variants as far transfer items. Near transfer items were primarily created by manipulation of superficial features of the original parent item (e.g. age, gender). Far transfer items were created by utilizing the underlying concept of the parent item and creating either a different answer involving a different clinical condition or different clinical decision that related to the concept. For example, an important conceptual understanding in medicine is that valvular heart disease has hemodynamic effects that differ depending on the valve affected. Peripheral examination findings can therefore be largely diagnostic of valvular disease. Given this, a parent item might describe peripheral examination findings associated with a particular cardiac valve disorder. Near transfer items would then describe a different patient with very similar peripheral examination findings (and the same cardiac valve disorder), while far transfer items might describe related peripheral signs associated with a different cardiac valve disorder. All items were reviewed independently by several content experts (for accuracy) and two item writing experts (to ensure items focused on application of knowledge and that none contained item-writing flaws).

For each parent item, three feedback variations were developed:

1. The baseline right/wrong feedback condition was a simple presentation of whether the question was answered correctly or incorrectly, and identification of which option was correct;
2. The response-oriented feedback provided brief explanations for why each option was correct or incorrect without emphasizing any underlying conceptual schemas for clinical reasoning;
3. The conceptually focused feedback described the pathophysiological or biomedical principles that are fundamental to the patient problem represented in the item and articulated the conceptual schemas necessary for solving clinical problems related to the same underlying concept.

### Procedures

Tests were delivered via an online survey tool (Qualtrics) in Toronto and using a bespoke iPad app in Melbourne. All tests were sat under exam conditions. The initial 2‑hour test was delivered in two stages: 1) presentation of 17 parent items as a block followed by presentation of feedback on all those items, again as a block, and 2) an immediate post-test presentation of 34 near and far transfer items. The follow-up retention test at 1 week contained 34 different near and far transfer items with 1.5 h allowed for test completion. Students were advised that the times allowed were considerably longer than the time usually allocated per item, and they should not feel the need to rush.

### Analysis

Our analysis was by ANOVA with item sets and items nested within item sets as random factors, and feedback type, transfer type, parent score, and occasion as fixed factors. The dependent measure was the logit-transformed *p*-value (proportion correct scores) for each item (i.e. the overall performance on each item when it was presented under each of the feedback and transfer type conditions). In our within-subjects design, a clear confound from analyzing the raw performance of each participant is the varying difficult levels of the items nested within item sets. Using the logit-transformed *p*-values allows us to control for confounding due to item difficulty. Prior to the final analysis, we also analyzed site differences and, finding none, we pooled all participants together (see Results). Further post-hoc analyses utilized the least significant difference (LSD) correction to control the experiment-wise error rate. Effect sizes were calculated using Cohen’s d.

## Results

Sixty-four participants were recruited into the study: 41 at Melbourne and 25 at Toronto. Two of the 25 Toronto students did not complete the final assessment. Our initial analysis revealed that that there was no significant site effect (*F* (1, 20.9) = 1.35, *p* < 0.26) with average scores of 53% for Toronto participants and 57% for Melbourne across both test occasions.

### Feedback reading times

Median times for responding to parent items and median time for viewing feedback provided during the initial test occasion are presented in Tab. 2. As expected, when individuals were exposed to conceptually focused feedback, they took significantly longer to read the feedback than when exposed to the other two types of feedback (*F* (2,694) = 182.3, *p* < 0.0001). Post-hoc testing confirmed that the participants spent significantly longer engaging with conceptually focused feedback than with either response-oriented or right/wrong feedback.

**Table 2:** Interaction (answer and reading) times for parent items overall and by correct and incorrect responses with comparison by subsequent feedback type (time in seconds)

	Median answer time (SD)	Median answer time when correct	Median answer time when incorrect	Median feedback reading time (SD)	Median feedback time when correct	Median feedback time when incorrect
Right/wrong	87 (44)	86	91	3 (5)	2	5
Response oriented	91 (45)	94	84	25 (24)	18	31
Conceptually focused	90 (45)	94	83	52 (49)	48	55

### Effect of feedback type

Analysis of the logit-transformed *p*-values revealed a significant effect of feedback type (*F* (2,330) = 7.4, *p* < 0.001) with mean scores on items following right/wrong feedback of 51.3%, following response-oriented feedback of 56.3%, and following conceptually focused feedback of 59.4%. This analysis used immediate post-test and 1‑week retention test data. Post-hoc testing confirmed that the response-oriented and conceptually focused feedback conditions were superior to right/wrong feedback with effect sizes of 0.2 and 0.4, respectively. As such, there are statistically significant and moreover meaningful differences between feedback formats. This has important implications for post-assessment feedback in formative contexts.

### Transfer effects

**Fig. 1:**
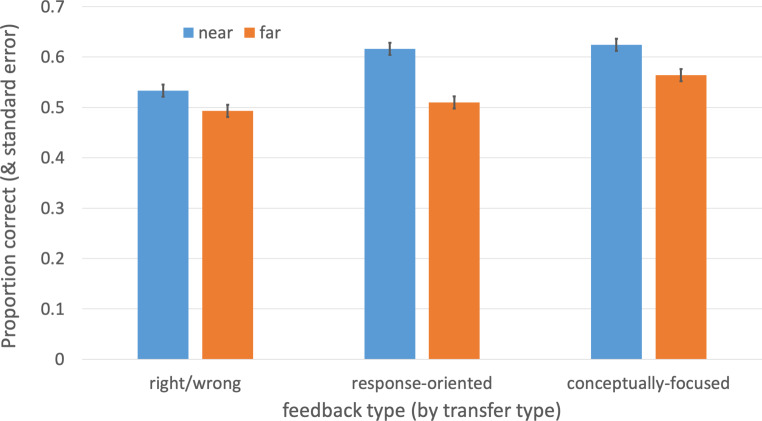
Proportion correct for near and far transfer items as a function of feedback type

Students generally scored higher on near transfer questions compared with the far transfer questions on both test occasions. Our analysis showed a significant effect of transfer type (*F* (1,330) = 15.61, *p* < 0.0001) with mean overall scores of 59.1% for near transfer and 52.2% for far transfer items. Moreover, we failed to detect significant effects of parent score (initially getting the item correct), (*F* (1, 330) = 1.35, *p* > 0.05) or test occasion (*F* (1,330) = 1.4, *p* > 0.05). No other interactions were significant in the analysis. We did not detect a significant interaction between transfer and feedback types (*F* (2,330) = 1.28, *p* < 0.277). That is, different forms of feedback had similar effects across both near and far transfer items (i.e. students improved more on near transfer items than for far transfer items for all forms of feedback – right/wrong, response-oriented, and conceptually focused). The results are presented graphically in Fig. 1.

### Exploratory analysis

We conducted further exploratory analyses to examine the impact of feedback type with the other factors in our study. While there was no significant three-way interaction among feedback type, transfer type, and parent score (*F* (2,330) = 0.616, *p* < 0.541), we detected an interesting pattern of results. Students receiving conceptually focused feedback on parent items performed better when subsequently answering a related far transfer item when the initial item was answered incorrectly (Tab. 3). Right/wrong feedback performance on far transfer was lower when the parent item was answered incorrectly. Pairwise comparisons showed that difference between right/wrong and conceptual feedback for far transfer when the item was answered incorrectly was a medium effect size (Cohen’s D = 0.6); a head-to-head comparison of means showed that this difference crossed the significance threshold, though the parent interaction was non-significant.

**Table 3:** Mean score (percentage correct) on near- and far-transfer items after parent item answered correctly or incorrectly by feedback type

		Near transfer (mean)	Far transfer (mean)
Parent item answered incorrectly	Right/wrong	60	46
	Response oriented	60	52
	Conceptually focused	61	59
Parent item answered correctly	Right/wrong	56	53
	Response oriented	63	50
	Conceptually focused	64	54

## Discussion

Our study aimed to test the efficacy of three different post-formative MCQ feedback formats on the transfer of knowledge to near and far transfer problems. Using a within-subjects design, we explored the effect of exposure to each type of feedback across 17 parent items and subsequent near and far transfer items at immediate and delayed testing (1 week later). Our analysis demonstrated that response-oriented and conceptually focused feedback was superior to traditional right/wrong feedback for both types of transfer tasks. However, there was no statistically significant difference between response-orientated and conceptually focused groups on near or far transfer problems, nor any differences in performance between our initial test occasion and the follow-up retention test 1 week later. As with most studies of transfer, participants’ near transfer scores were higher than far transfer scores.

Overall, our results suggest that elaborating on feedback beyond simple right/wrong presentation can enhance transfer to new problems and support assessment for learning, at least in the short term. Our experimental findings showed different patterns of engagement as measured by the time spent engaging with feedback across the three feedback formats, as well as for correct vs. incorrect responses to parent items. These results suggest that the experimental protocol was adhered to by participants (i.e. students spent time engaging with the different types of feedback and more time with the more detailed feedback types) and that, in general, students engaged to a greater degree with the more detailed conceptually focused feedback.

Despite the lack of statistical significance, our results suggest some interesting patterns that appear worthy of further exploration. More specifically, when participants answered a parent item incorrectly and received conceptually focused feedback, the subsequent performance on a related far transfer item was superior when compared with right/wrong feedback. There are a number of post-hoc explanations for this effect but further larger sample studies – participants and items – are necessary before this effect is confirmed.

Our results build on a larger literature on formative assessment feedback within the field of cognitive psychology [14, 15]. Generally, these studies show the positive effect of feedback, though in most circumstances the feedback format takes the form of ‘knowledge of results’, i.e. right or wrong. Moreover, most of these studies focused on retention of knowledge. The question of how feedback can be elaborated to enhance transfer and other more advanced learning outcomes merits further investigation and elaboration. Conceptual feedback as defined in this experiment is best viewed as only one of several approaches to this goal.

As with all studies, our experiment had some limitations. Our assessment items, though created by experienced item writers, showed varied item difficulties and may have attenuated some of our findings. Wider sampling of items and concept areas is desirable to understand the generality of the effects. Additionally, the areas of clinical reasoning we identified as challenging for early clinical learners and the subsequent selection of underlying foundational concepts were achieved via consensus. Another group of experts may pose other approaches to identifying foundational concepts and so further study and validation of the concepts used as the basis of conceptual feedback is recommended. Our experiment also did not control for individual prior knowledge (as we did not have permission to access this information about our participants) – only through selection of students in pre-clinical or very early clinical training. Lastly, the time interval for retention and transfer was relatively brief: 1 week. While this is a common time delay in experimental studies, longer delays are desirable to test the permanence of the effects found in this experiment.

## Conclusion

Practice testing is an increasingly common part of curricula. To fully leverage the benefits of test-enhanced learning, educators should consider elaborating on post-assessment feedback beyond just providing knowledge of right and wrong. Future research should investigate the roles of feedback format and content to promote and sustain transfer of learning.
